# Survival of patients with rare diseases: a population-based study in Tuscany (Italy)

**DOI:** 10.1186/s13023-021-01907-0

**Published:** 2021-06-14

**Authors:** Francesca Gorini, Alessio Coi, Lorena Mezzasalma, Silvia Baldacci, Anna Pierini, Michele Santoro

**Affiliations:** 1 Unit of Epidemiology of Rare Diseases and Congenital Anomalies, Institute of Clinical Physiology, National Research Council, via Moruzzi 1, 56124 Pisa, Italy; 2 Fondazione Toscana “Gabriele Monasterio”, Pisa, Italy

**Keywords:** Rare disease, Survival, Mortality risk, Disease registry

## Abstract

**Background:**

Rare diseases (RDs) encompass a heterogeneous group of life-threatening or chronically debilitating conditions that individually affect a small number of subjects but overall represent a major public health issue globally. There are still limited data on RD burden due to the paucity of large population-based epidemiological studies. The aim of this research was to provide survival estimates of patients with a RD residing in Tuscany, Italy.

**Methods:**

Cases collected in the Rare Diseases Registry of Tuscany with diagnosis between 1st January 2000 and 31th December 2018 were linked to the regional health databases in order to retrieve information on mortality of all subjects. Survival at 1, 5 and 10 years from diagnosis with 95% confidence intervals (CI) was estimated by sex, age class, nosological group and subgroup using the Kaplan–Meier method. The effect of sex, age and period of diagnosis (years 2000–2009 or 2010–2018) on survival was estimated using Cox proportional hazards regression.

**Results:**

Survival at 1, 5 and 10 years from diagnosis was 97.3%, 88.8% and 80.8%, respectively. Respiratory diseases and peripheral and central nervous system disorders were characterized by the lowest survival at 5 and 10 years. Despite a modest higher prevalence of RDs among females (54.0% of the total), male cases had a significant increased risk of death (hazard ratio, HR 1.48, 95% CI 1.38–1.58). Cases diagnosed during 2010–2018 period had a risk of death significantly lower than those diagnosed during 2000–2009 (HR 0.81, 95% CI 0.82–0.96), especially for immune system disorders (HR 0.48, 95% CI 0.26–0.87), circulatory system diseases (HR 0.61, 95% CI 0.45–0.84) and diseases of the musculoskeletal system and connective tissue (HR 0.64, 95% CI 0.49–0.84).

**Conclusions:**

An earlier diagnosis as well as the improvement in the efficacy of treatment resulted in a decreased risk of death over the years for specific RDs. The linkage between a population-based registry and other regional databases exploited in this study provides a large and accurate mass of data capable of estimating patients’ life-expectancy and increasing knowledge on the collective burden of RDs.

**Supplementary Information:**

The online version contains supplementary material available at 10.1186/s13023-021-01907-0.

## Background

Rare diseases (RDs) encompass a heterogeneous group of health conditions which, while individually affect a small number of subjects compared to other diseases prevalent in the general population, on the whole represent a major public health issue at the global level [1, 2]. In the European Union, any life-threatening or chronically debilitating condition affecting less than 5 people in 10,000 is considered rare, whereas a RD in the United States is any condition that affects fewer than 200,000 people, which corresponds to a prevalence of about 1 in 1630 people [3]. Other national jurisdictions set prevalence thresholds from 5 cases per 100,000 for Korea to 76 cases per 100,000 people for China [4]. With an estimate that at least 1 out of 16 people suffers from a RD, more than 470 million people worldwide, of whom 46.5 million in Europe and 20.5 million in the United States, could be affected by one of 6000–8000 RDs [3, 5]. Actually, the exact number of RDs is unknown and depends on the source, and is meanwhile increasing due to new RDs reported periodically in the medical literature [4, 6]. The Orphanet (www.orpha.net) is an information portal that contains data on 6172 unique RDs, of which 71.9% are classified as genetic, involving either hereditary or de novo mutations [7].

Despite their great diversity, the majority of RDs are classified as severe to very severe, have no cure, and can result in serious consequences including premature deaths in infancy and shortened lifespan in adults, as well [8, 9]. According to data from the European Conference on Rare Diseases hold in 2005 and based on 323 RDs, only 37.5% of diseases are characterized by a normal lifespan [9]. Consistent with previous findings, a study analyzing 430 Mendelian disorders found that 35% of them have a normal life expectancy, and 16 to 29% of these diseases are associated with symptoms ranging from mild to severe, respectively [10]. Of note, the number of pediatric RD patients who experience the transition to adulthood is increasing and represents a non-negligible proportion of the RD population [11].

Public awareness of RDs has been progressively growing in recent years due to the ever-closer collaboration between patient associations, researchers and clinicians, politicians and industry [2]. The burden of RDs on society translates into high mortality and disability rates, high rates of hospital admissions and long-term care, and consequent elevated costs for the national health-care systems or, when the economic coverage for treatment is not complete, for the patients [3]. Additionally, patients with RDs often face difficulties related to orientation and medical pathway to reach a correct diagnosis and subsequently an adequate follow-up, both medical and social [12]. For many RDs, cause is not yet identified and molecular and pathophysiological mechanisms are unknown and epidemiological data collection is scarce or even unavailable [8, 13], making it difficult both to estimate the overall burden of RDs and address health-care planning to evaluate the economic and societal effects of RDs [2, 14].

As many RDs individually affect very few people, the major concern of the RD community is the absence of reliable epidemiological data on the prevalence, incidence and life expectancy of RDs at the national and global level in order to implement public health measures and improve RD diagnosis and treatment [15].

To the best of our knowledge, studies on survival of people affected by a RD using population-based data, have not been published so far. The aim of this population-based study was to provide the survival estimates of RD patients residing in Tuscany, Italy.

## Methods

In this retrospective cohort study, the monitored population includes all cases with a RD residing in Tuscany, an Italian region of 3,701,343 inhabitants (source: Italian National Institute of Statistics at 1st January 2018). Cases diagnosed between 1th January 2000 and 31th December 2018 with one of the RDs reported in the list of the Italian law (Decree of the President of the Council of Ministers, 01/2017) (Additional file 1), which includes 804 diseases divided into 16 nosological groups and 17 subgroups, and identified by a specific six-digit code for exemption from co-payment, were collected from the population-based Rare Diseases Registry of Tuscany. The registry has been active since 2005 and is based on a regional network of health centres, some of which are centres of expertise [16].

Cases with RDs extracted from the Rare Diseases Registry of Tuscany were linked to the regional health databases, i.e., mortality database, hospital discharge records, and Registry Office database, through a unique anonymous identification number. For each case, the date of diagnosis reported in the Rare Diseases registry was used. Information on mortality was retrieved from the regional mortality and hospital discharge databases. The vital status of the subjects as at 31st December 2018 and the number of cases emigrated from the region during the follow-up period were ascertained through the Registry Office database.

Since the risk of death for congenital anomalies is high in early life [17, 18], and the Rare Diseases Registry of Tuscany is not suitable for collecting cases with rare congenital anomalies diagnosed in the first weeks of life [16], the cases of the nosological group ‘congenital anomalies, chromosomal aberrations and genetic syndromes’, were included only if diagnosed beyond the first month of life.

Survival estimates at 1, 5 and 10 years from diagnosis were calculated by sex, age class (< 18 years, 18–65 years, and ≥ 65 years), nosological group and subgroup using the Kaplan–Meier method. For subjects suffering from two or more diseases of the same nosological group, the RD diagnosed first was considered. Subjects with two or more disease of different nosological group were counted once when estimating the survival on the overall cohort. The effects of sex, age and period of diagnosis (2010–2018 vs. 2000–2009 period) on survival were estimated using Cox proportional hazards regression model and hazard ratios (HRs) with 95% confidence intervals (CI) were calculated. The data were analyzed with Stata, version 16 [19]. Two-sided p-value < 0.05 was considered statistically significant in all analyses of this study.

## Results

A total of 23,671 cases, diagnosed during 2000–2018 and endowed of a regional anonymous identification number necessary for the linkage with the regional databases, represent the study cohort.

In the first study period (2000–2009), 8996 cases received a diagnosis of RD, while the remaining patients (62.0%) were diagnosed with a RD in the 2010–2018 period.

We observed a modest higher proportion of females (12,787 cases corresponding to 54.0% of the total), especially in ‘diseases of musculoskeletal and connective tissue’ (81.1%) and ‘endocrine diseases’ (73.3%). The median age at diagnosis was 44.3 years and, with regards to the age distribution, 5026 cases (21.2%) were pediatric patients (< 18 years), 12,609 (53.3%) were aged between 18 and 64 years, and 6036 (25.5%) were 65 and over.

**Fig. 1:**
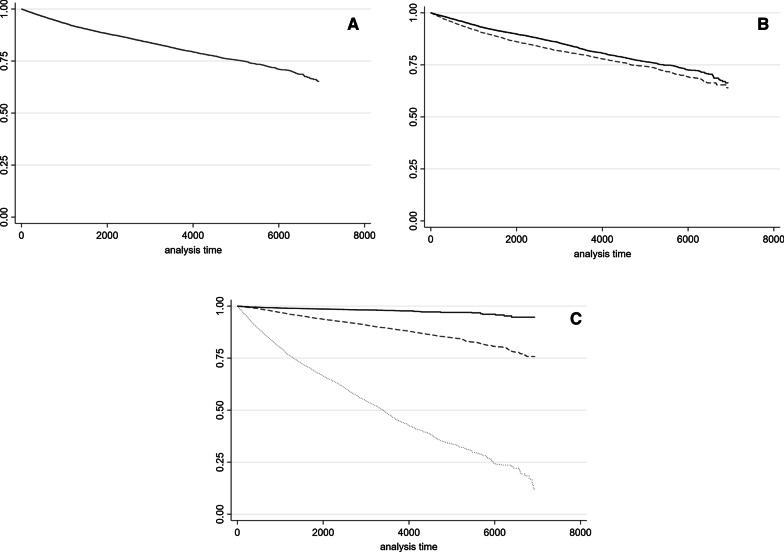
Kaplan–Meier survival estimates for the total of cases (**A**), by sex (**B**: solid line, females; dashed line, males) and by age class (**C**: solid line, < 18 years; dashed line 18–64 years; dotted line ≥ 65 years)

The total person-time of the cohort was 156,183 years and, during the study period, 3421 deaths (14.41%) were recorded, of which 1696 were females and 1725 were males. In Fig. 1A–C, survival curves for overall cases were reported by sex and age class.

Survival at 1, 5 and 10 years from diagnosis was 97.3% (95% CI 97.1–97.5), 88.8% (95% CI 88.4–89.3) and 80.8% (95% CI 80.1–81.5), respectively (Fig. 1A, Table 1), with females showing an expectancy life of 90.6% (95% CI 90.0–91.1 and males of 87.2% (95% CI 86.5–87.8) (Fig. 1B). Survival at 5 years was 98.7% (95% CI 97.2–98.3), 94.2% (95% CI 93.7–94.6), and 68.4% (95% CI 67.0–69.7) for subjects < 18, between 18 and 64, and ≥ 65 years, respectively (Fig. 1C).

**Table 1:** Survival of the cohort at 1, 5 and 10 years from diagnosis for the total number of cases and for each nosological group

	CASES (n)	Age at diagnosis (mean ± standard deviation)	% males	1 year	95% CI	5 years	95% CI	10 years	95% CI
Peripheral and central nervous system disorders	5928	53.0 ± 21.3	51.6	0.953	0.948–0.959	0.796	0.785–0.807	0.679	0.664–0.694
Congenital anomalies, chromosomal aberrations and genetic syndromes^a^	2377	17.4 ± 18.2	51.5	0.998	0.995–1.000	0.984	0.977–0.989	0.961	0.949–0.970
Circulatory system diseases	2177	55.4 ± 19.7	35.2	0.982	0.975–0.987	0.917	0.903–0.929	0.800	0.776–0.821
Diseases of the musculoskeletal system and connective tissue	1826	57.3 ± 15.5	18.9	0.973	0.964–0.980	0.892	0.875–0.907	0.768	0.740–0.794
Metabolic diseases	1773	36.7 ± 28.0	58.2	0.962	0.951–0.963	0.899	0.883–0.914	0.845	0.822–0.865
Respiratory diseases	1639	60.1 ± 16.4	57.3	0.947	0.934–0.957	0.719	0.687–0.749	0.565	0.520–0.608
Diseases of the skin and subcutaneous tissue	1413	58.9 ± 22.3	43.9	0.949	0.936–0.960	0.811	0.786–0.833	0.715	0.677–0.748
Diseases of the blood and blood-forming organs	1361	27.9 ± 22.3	48.6	0.992	0.986–0.996	0.972	0.960–0.981	0.962	0.947–0.973
Immune system disorders	1331	38.5 ± 23.0	38.8	0.998	0.994–1.000	0.969	0.956–0.979	0.903	0.875–0.925
Disorders of the eye and adnexa	1301	37.5 ± 18.6	57.3	0.998	0.993–0.999	0.989	0.981–0.994	0.964	0.947–0.975
Endocrine diseases	1036	24.1 ± 20.5	26.7	1.000	–	0.988	0.976–0.994	0.957	0.933–0.973
Neoplasms	987	31.5 ± 22.7	45.2	0.986	0.977–0.992	0.944	0.926–0.958	0.923	0.899–0.941
Diseases of the genitourinary system	489	43.0 ± 20.5	47.8	0.996	0.983–0.999	0.989	0.971–0.996	0.954	0.907–0.977
Digestive disorders	257	51.1 ± 21.4	53.7	0.968	0.936–0.984	0.896	0.841–0.933	0.818	0.720–0.884
1infectious and parasitic diseases	13	47.2 ± 19.7	53.8	1.000	–	0.909	0.508–0.987	0.909	0.508–0.987
**Total**	**23,671**	**44.3 ± 25.0**	**46.0**	**0.973**	**0.971**–**0.975**	**0.888**	**0.884**–**0.893**	**0.808**	**0.801**–**0.815**

Bold is provided for total rare disease survival results

^a^Only cases diagnosed beyond 1 month. The total number of cases shown in the table does not refer to the sum of cases for each nosological group. Survival estimates of neonatal morbidities of perinatal origin not shown as only 6 cases were reported. *95% CI* 95% confidence interval

Survival at 1, 5 and 10 years from the first diagnosis for each nosological group and the entire cohort was reported in Table 1. Patients suffering from ‘respiratory diseases’, ‘peripheral and central nervous system (CNS) disorders’ and ‘diseases of the skin and subcutaneous tissue’ had the worst life expectancy, with a survival of 56.5%, 67.9%, and 71.5% respectively, after 10 years. Cases with ‘circulatory system diseases’, ‘digestive disorders’, ‘metabolic diseases’ and ‘diseases of the musculoskeletal system and connective tissue’ had a comparable survival at 5 years from diagnosis (range 89.2–91.7%), although patients with ‘metabolic diseases’ experienced a higher survival at 10 years (84.4% vs. range 76.8–81.8% for the others). As for diseases belonging to the group defined as ‘neoplasms’, survival at 5 and 10 years was 94.4 and 92.3%, respectively, while subjects with ‘immune system disorders’ had a survival of 90.3% at 10 years from diagnosis. At 1 year, only subjects with ‘endocrine diseases’ and with ‘infectious and parasitic diseases’ showed a 100% survival. Patients with a RD from the groups of ‘endocrine diseases’, ‘diseases of the blood and blood-forming organs’ and’diseases of the genitourinary system’, ‘disorders of the eye and adnexa’, or ‘congenital anomalies, chromosomal aberrations and genetic syndromes’ had the highest life expectancy both at 5 years (range 97.2–98.9%) and 10 years (range 95.4–96.4%) from diagnosis.

Survival estimates for the subgroups of metabolic diseases were reported in Table 2. ‘Disorders of protein metabolism and transport’ were characterized by the lowest survival among the metabolic diseases (40.0% at 10 years), but this may be due to the higher mean age observed at diagnosis (65.4 vs. 36.7 years of the group). Approximately 15% and 20% of patients with ‘lysosomal storage diseases’ did not survive at 5 and 10 years from diagnosis, respectively. Patients with a RD belonging to the subgroups ‘disorders of mitochondrial metabolism’ and ‘disorders of metal metabolism and transport’, had a survival ranging from 96.6 to 97.7% at 5 years and from 91.5 to 92.3% at 10 years. Subjects who suffered from a disease of the subgroup ‘disorders of vitamin and non-protein cofactor absorption and transport’ presented the highest survival (98.0%, CI 95% 92.2–99.5) at 5 years.

**Table 2:** Survival of the cohort at 1, 5 and 10 years from diagnosis for the subgroup of metabolic diseases

	Cases (n)	1 year	95% CI	5 years	95% CI	10 years	95% CI
Disorders of metal metabolism and transport	404	0.995	0.980–0.999	0.977	0.954–0.988	0.923	0.885–0.949
Disorders of protein metabolism and transport	320	0.848	0.802–0.884	0.577	0.506–0.642	0.400	0.310–0.487
Disorders of mitochondrial metabolism	304	0.986	0.962–0.995	0.966	0.931–0.983	0.915	0.849–0.953
Lysosomal storage diseases	150	0.966	0.920–0.986	0.855	0.774–0.908	0.803	0.710–0.870
Disorders of vitamin and non-protein cofactor absorption and transport	111	0.990	0.934–0.999	0.980	0.922–0.995	0.980	0.922–0.995
Other metabolic diseases^a^	485	0.985	0.969–0.993	0.971	0.950–0.984	0.948	0.914–0.968

^a^Group of metabolic diseases not attributable to the others listed in the table. 95*% CI* 95% confidence interval

Cox proportional hazards regression showed that, for all cases, males had a significantly increased risk of death than females (HR: 1.48, 95% CI: 1.38–1.58), and each additional year at diagnosis was associated with a 7% risk of dying (Table 3). In the 2010–2018 period, the risk of death was 11% lower than in the 2000–2009 period (HR 0.89, 95% CI 0.82–0.96).

**Table 3:** Effect of sex, age at diagnosis, and period of diagnosis on mortality risk for total cases and by nosological group

	Sex		Age at diagnosis		Period of diagnosis	
	HR^a^	95% CI	HR^b^	95% CI	HR^c^	95% CI
Neoplasms	1.43	0.90–2.29	**1.04**	1.03–1.06	0.79	0.45–1.36
Endocrine diseases	**2.01**	1.02–3.94	**1.09**	1.06–1.13	1.16	0.41–3.26
Metabolic diseases	1.08	0.82–1.42	**1.05**	1.04–1.06	1.20	0.88–1.62
Immune system disorders	**2.13**	1.35–3.35	**1.06**	1.05–1.08	**0.48**	0.26–0.87
Diseases of the blood and blood-forming organs	0.99	0.52–1.88	**1.07**	1.05–1.09	0.93	0.47–1.86
Peripheral and central nervous system disorders	1.08	0.98–1.20	**1.07**	1.06–1.07	0.96	0.86–1.08
Disorders of the eye and adnexa	**1.83**	1.04–3.19	**1.11**	1.09–1.14	0.81	0.33–1.96
Circulatory system diseases	**1.50**	1.20–1.88	**1.09**	1.08–1.10	**0.61**	0.45–0.84
Respiratory diseases	**1.79**	1.43–2.23	**1.08**	1.07–1.09	0.80	0.62–1.03
Digestive disorders	0.62	0.30–1.31	**1.12**	1,07–1,16	0.95	0.33–2.76
Diseases of the genitourinary system	2.48	0.84–7.29	**1.12**	1.06–1.18	0.34	0.06–1.90
Diseases of the skin and subcutaneous tissue	**1.53**	1.22–1.93	**1.12**	1.10–1.14	0.84	0.64–1.10
Diseases of the musculoskeletal system and connective tissue	**1.63**	1.24–2.14	**1.10**	1.09–1.12	**0.64**	0.49–0.84
Congenital anomalies, chromosomal aberrations and genetic syndromes^a^	**1.63**	1.03–2.58	**1.05**	1,04–1,06	0.75	0.40–1.41
**Total**	**1.48**	**1.38–1.58**	**1.07**	**1.07–1.08**	**0.89**	**0.82–0.96**

^a^Only cases diagnosed beyond 1 month. For the nosological group of infectious and parasitic diseases the small number of cases did not allow to calculate HRs. Females were the reference group in the analysis by sex; the 2000–2009 period was the reference group and was compared to 2010–2018 period in the analysis by period of diagnosis. As for congenital anomalies, chromosomal aberrations and genetic syndromes, cases diagnosed by one month of age were excluded. *HR* hazard ratio, *95% CI* 95% confidence interval

^a^HRs were adjusted for age and period of diagnosis

^b^HRs were adjusted for sex and period of diagnosis

^c^HRs were adjusted for sex and age at diagnosis. Statistically significant differences are reported in bold

A significant increase in the risk of death was observed among males, from ‘immune system disorders’, ‘endocrine diseases’, ‘disorders of the eye and adnexa’, ‘respiratory diseases’, ‘diseases of the musculoskeletal system and connective tissue’, ‘congenital anomalies, chromosomal aberrations and genetic syndromes’, ‘diseases of the skin and subcutaneous system’, and ‘circulatory system diseases’.

A significant reduction in mortality risk in the period 2010–2018 was found for ‘immune system disorders’, ‘circulatory system diseases’ and ‘diseases of the musculoskeletal system and connective tissue’.

Cox regression analysis for the subgroup of metabolic diseases did not produce significant differences between males and females (Table 4). A significant increased risk of mortality with increasing age at diagnosis was observed for almost all the subgroups, with the exception of ‘lysosomal storage diseases’ and ‘disorders of vitamin and non-protein cofactor absorption and transport’. Furthermore, no significant differences in mortality risk were observed between the 2000–2009 and the 2010–2018 periods.

**Table 4:** Effect of sex, age and period of diagnosis on mortality risk in the subgroups of metabolic diseases

	Sex		Age at diagnosis		Period of diagnosis	
	HR^b^	95% CI	HR^c^	95% CI	HR^d^	95% CI
*Metabolic diseases*						
Disorders of mitochondrial metabolism	3.12	0.99–9.80	**1.05**	1.02–1.09	0.67	0.20–2.25
Lysosomal storage diseases	1.21	0.56–2.61	0.99	0.96–1.01	1.04	0.39–2.79
Disorders of vitamin and non-protein cofactor absorption and transport	5.87	0.96–35.98	1.05	1.00–1.11	0.17	0.02–1.54
Disorders of metal metabolism and transport	3.14	0.96–10.30	**1.09**	1.03–1.15	0.87	0.31–2.43
Disorders of protein metabolism and transport	0.72	0.049–1.04	**1.05**	1.04–1.07	0.96	0.61–1.50
Other metabolic diseases^a^	2.04	0.81–1.17	**1.04**	1.03–1.07	0.70	0.26–0.94

^a^Group of metabolic diseases not attributable to the others listed in the table. Females were the reference group in the analysis by sex; the 2000–2009 period was the reference group and was compared to 2010–2018 period. *HR* hazard ratio; *95% CI* 95% confidence interval

^b^HRs were adjusted for age and period of diagnosis

^c^HRs were adjusted for sex and period of diagnosis

^d^HRs were adjusted for sex and age at diagnosis. Statistically significant differences are reported in bold

## Discussion

The current study provided survival profiles of RD patients, based on data from a population-based registry surveilling more than 800 RDs in a geographic area of about 3,700,000 inhabitants.

Our results showed that overall survival in RD subjects was 88.8% and 80.8% at 5 and 10 years after diagnosis, respectively. Males and especially the elderly experienced a shorter life expectancy, in accordance with a previous Italian study observing that the highest fatality rate calculated on data from a RD population-based registry, was among the subjects over 65 [20].

The ‘peripheral and CNS disorders’ and ‘respiratory diseases’, representing respectively 25.0 and 6.9% of total cases, were the nosological groups with the lowest survival. This result could be attributable to the most frequent diseases of these groups, amyotrophic lateral sclerosis (ALS) (21.7% of cases in ‘peripheral and CNS disorders’) and sarcoidosis (47.2% of cases in ‘respiratory diseases’), both characterized by a high fatality rate. In fact, recent literature has reported that the vast majority of patients with ALS have a mean or median survival time between 24 and 50 months from symptoms onset to death or invasive respiratory support [21]. Our findings on ‘peripheral and CNS disorders’ are in agreement with Mazzucato and co-authors who found a very high fatality rate associated with this group of diseases in the monitored population [20]. Likewise, sarcoidosis is a major contributor to the ‘respiratory disease’ group. A study conducted in a cohort of 452 American patients with diagnosis of sarcoidosis revealed that the overall mortality from sarcoidosis was 3.9% at 5 years, reaching 9.0% at 10 years [22]. The observed low 10-year survival estimate for overall ‘respiratory diseases’ could also be explained by the high mean age at diagnosis observed for this nosological group (60.1 years).

The ‘disease of the skin and subcutaneous disease’ group also had a low survival (81.1% at 5 years and 71.5% at 10 years) compared to other groups, and this can be interpreted in a higher mean age at diagnosis (58.9 years) and in the presence of a large number of individuals with pemphigus (15.6% of cases) and bullous pemphigoid (30.0% of cases), which are often characterized by a severe prognosis. Indeed, a retrospective hospital-based cohort study carried out on 108 Romanian patients with pemphigus vulgaris, demonstrated that an age of onset ≥ 65 years and the presence of coronary heart disease at diagnosis were independent risk factors associated with poor survival [23]. According to a retrospective study performed on eighty-seven American residents diagnosed with bullous pemphigoid, the estimated overall survival rates were 47%, 23%, and 21% at 4, 6 and 10 years after diagnosis, respectively [24].

From the analysis of the subgroups of metabolic diseases, a poor life expectancy was also observed among patients suffering from ‘disorders of protein metabolism and transport’ and was likely attributable to primary systemic amyloidosis, which is the most frequent disease in the subgroup (76.1% of cases) and with a recognized low survival. Consistently, Kumar et al. reported a median survival of patients with amyloidosis ranging from 12 to 18 months, depending on the number of organs involved and the severity of the disease [25].

As regards the group of ‘congenital anomalies, chromosomal aberrations and genetic syndromes’, we found a high life expectancy (96.1% at 10 years). It was somewhat predictable, since the regional Registry of Rare Diseases is not sensitive to collect cases diagnosed in the first weeks of life [16], which is the timeframe with the highest mortality from congenital anomalies. To avoid selection bias, we decided to include only patients diagnosed with rare congenital anomalies beyond the first month of life. Hence, the survival value provided in our study could be interpreted as an estimate of the survival of patients with rare congenital anomalies given that the child survived to the first month. On the other hand, the registry of congenital anomalies of Tuscany (www.rtdc.it), whose network is based on maternity units, is the most sensitive tool for estimating the survival of children born with a rare congenital anomaly. These factors partly explain why in our study we observed an elevated age at diagnosis (mean age of 17.4 years) for this RD group. Another reason that clarifies an average age in the adolescence range is the presence of congenital anomalies characterized by variable phenotypic expression, which can remain asymptomatic and often are diagnosed later in life (e.g., Arnold–Chiari syndrome, Klippel–Feil syndrome) [26, 27].

Overall and as expected, an older age at diagnosis negatively impacted the mortality risk of both RDs as a whole and that of nosological groups and subgroups when estimates were possible. The association between sex and survival was highly significant for the total of cases and especially for specific nosological groups, possibly as a consequence of the increased probability of complications among males, which could lead to an early fatal outcome [28]. For this reason, ad hoc studies on specific disease are needed to provide insights into survival differences.

The current study also showed that, for some nosological groups, the risk of death has decreased in recent years. These findings may be owing to better overall patient management, as well as improved timeliness of diagnosis in the last few years. Nonetheless, due to the general lack of data, in particular wide population-based studies in the literature, the comparison of our results with previous studies, is only possible for specific diseases. For instance, referring to the drop of 46% in the risk of death observed in ‘diseases of the musculoskeletal system and connective tissue’ between the 2000–2009 and 2010–2018 periods, an analysis on systemic sclerosis, the most important disorder of autoimmune rheumatological disease, observed a reduction in the mortality rate from 2003 to 2016 in the United States [29], possibly due to the improved early diagnosis and consequently treatment, which decrease the impact of complications and comorbidities [28].

### Strengths

To date, no other studies have analyzed the survival of patients with a RD collected in a population-based registry. It is well known that, while patient registries are considered key instruments to increase understanding of the natural history of rare conditions and improve clinical research, patient care and disease management, but are not representative of the residing population, population-based registries include all existing patient cases and use the data for burden measures, disease descriptive epidemiology, and risk factors, therefore representing an essential tool for public health surveillance [30].

In addition, the Rare Diseases Registry of Tuscany is based on a consolidate network that collects and validates data on about 800 different RDs using standardized criteria of inclusion.

Furthermore, we used a multi-database approach that integrates information from the Rare Diseases Registry of Tuscany with other health regional databases, thus providing data to evaluate the outcomes and burden of RDs in order to increase knowledge on the collective impact of RDs. The methodological approach here applied contributes to ameliorate the surveillance of RDs with accurate and reliable epidemiological indicators based on a large cohort of cases and stimulates the study of outcomes for specific RDs or groups of RDs.

### Limitations

This study has some limitations. First of all, the RDs registered in the Rare Disease Registry of Tuscany are those surveilled by the Italian law (Decree of the President of the Council of Ministers, 01/2017) and equipped with a code of exemption from co-payment (see Additional file 1). The exemption code not always corresponds to a specific ORPHA code and the definition reported in Additional file 1 often refers to group or subgroup of similar diseases. Secondly, as reported above, the Rare Disease registry is not suitable for collecting cases of congenital anomalies in the first few weeks of life, therefore the survival estimates for this group are limited to cases diagnosed after the first month, and consequently higher than those expected for this group of RDs Besides, since the Rare Diseases Registry of Tuscany is active from 2005, the ascertainment of cases collected retrospectively for the previous years can be underestimated.

## Conclusion

This study analyzed the survival of patients with a RD collected in a population-based registry by applying a multi-database approach capable of providing accurate survival estimates. Survival at 5 and 10 years from diagnosis was 88.8% and 80.8%, respectively, considering all patients. Differences were found between nosological groups, with ‘respiratory diseases’ and ‘peripheral and CNS disorders’ characterized by the lowest survival at both 5 and 10 years. While a higher prevalence of cases was detected among females, male patients had a shorter lifespan for most nosological groups. As expected, an older age at diagnosis was significantly associated with an increased risk of mortality in all RD groups. In the 2010–2018 period, on the other hand, a significantly lower risk of death (− 11%) for the total number of cases was observed compared to the 2000–2009 period, in particular for the groups of ‘immune system disorders’, ‘circulatory system diseases’ and ‘diseases of the musculoskeletal system and connective tissue’, probably due to early diagnosis and/or more effective treatments.

Other epidemiological studies based on a large cohort of cases are advisable in order to improve knowledge on RDs and provide meaningful data to clinicians, researchers and policy makers.

## Supplementary Information

**Additional file 1.** **Table S1.** List of rare diseases endowed of an exemption code as defined by the Italian law and included in the study. For each nosological group, groups of diseases are reported in bold and, if available, specific diseases belonging to the groups are indicated.

## Data Availability

The data that support the findings of this study are available from Regione Toscana but restrictions apply to the availability of these data, which were used under license for the current study, and so are not publicly available. Data are however available from the authors upon reasonable request and with permission of Tuscany Region.

## References

[1] Posada de la Paz MP, Villaverde-Hueso A, Alonso V, János S, Zurriaga O, Pollán M, Abaitua-Borda I (2010). Rare diseases epidemiology research. Adv Exp Med Biol.

[2] Schieppati A, Henter JI, Daina E, Aperia A (2008). Why rare diseases are an important medical and social issue. Lancet.

[3] Ferreira CR (2019). The burden of rare diseases. Am J Med Genet A.

[4] Richter T, Nestler-Parr S, Babela R, Khan ZM, Tesoro T, Molsen E, Hughes DA, International Society for Pharmacoeconomics and Outcomes Research Rare Disease Special Interest Group (2015). Rare disease terminology and definitions—a systematic global review: report of the ISPOR Rare Disease Special Interest Group. Value Health..

[5] Lancet T (2020). Rare diseases need sustainable options. Lancet.

[6] Haendel M, Vasilevsky N, Unni D, Bologa C, Harris N, Rehm H, Hamosh A, Baynam G, Groza T, McMurry J, Dawkins H, Rath A, Thaxon C, Bocci G, Joachimiak MP, Köhler S, Robinson PN, Mungall C, Oprea TI (2020). How many rare diseases are there?. Nat Rev Drug Discov.

[7] Nguengang Wakap S, Lambert DM, Olry A, Rodwell C, Gueydan C, Lanneau V, Murphy D, Le Cam Y, Rath A (2020). Estimating cumulative point prevalence of rare diseases: analysis of the Orphanet database. Eur J Hum Genet.

[8] Valdez R, Ouyang L, Bolen J (2016). Public health and rare diseases: oxymoron no more. Prev Chronic Dis.

[9] EURORDIS, 2005. European Conference on Rare Diseases 2005 Report. https://www.eurordis.org/IMG/pdf/EN-ECRDtotal-2.pdf. Accessed 30 Jan 2021.

[10] Jimenez-Sanchez G, Childs B, Valle D, 2014. The effect of Mendelian disease on human health. In A. L. Beaudet, B. Vogelstein, K. W. Kinzler, S. E. Antonarakis, A. Ballabio, K. M. Gibson, & G. Mitchell (Eds.), The online metabolic and molecular bases of inherited disease (Vol. 1—Book, Section). New York, NY: The McGraw-Hill Companies Available at: https://ommbid.mhmedical.com/content.aspx?sectionid=225070731&bookid=2709&Resultclick=2. Accessed 22 March 2021.

[11] Mazzucato M, Visonà Dalla Pozza L, Minichiello C, Manea S, Barbieri S, Toto E, Vianello A, Facchin P (2018). The epidemiology of transition into adulthood of rare diseases patients: results from a population-based registry. Int J Environ Res Public Health.

[12] Puiu M, Dan D (2010). Rare diseases, from European resolutions and recommendations to actual measures and strategies. Maedica (Bucur).

[13] de Vrueh R, Baekelandt ERF, de Haan JMH. Priority medicines for Europe and the world: “a public health approach to innovation.” WHO Background Paper 6.19. Rare Diseases; 2013. https://www.who.int/medicines/areas/priority_medicines/BP6_19Rare.pdf. Accessed 26 Jan 2021.

[14] Walker CE, Mahede T, Davis G, Miller LJ, Girschik J, Brameld K, Sun W, Rath A, Aymé S, Zubrick SR, Baynam GS, Molster C, Dawkins HJS, Weeramanthri TS (2017). The collective impact of rare diseases in Western Australia: an estimate using a population-based cohort. Genet Med.

[15] Groft SC, Posada de la Paz M (2017). Rare diseases: joining mainstream research and treatment based on reliable epidemiological data. Adv Exp Med Biol.

[16] Coi A, Santoro M, Pierini A, Marrucci S, Pieroni F, Bianchi F (2017). Prevalence estimates of rare congenital anomalies by integrating two population-based registries in Tuscany, Italy. Public Health Genom.

[17] Boyle B, Addor MC, Arriola L, Barisic I, Bianchi F, Csáky-Szunyogh M, de Walle HEK, Dias CM, Draper E, Gatt M, Garne E, Haeusler M, Källén K, Latos-Bielenska A, McDonnell B, Mullaney C, Nelen V, Neville AJ, O'Mahony M, Queisser-Wahrendorf A, Randrianaivo H, Rankin J, Rissmann A, Ritvanen A, Rounding C, Tucker D, Verellen-Dumoulin C, Wellesley D, Wreyford B, Zymak-Zakutnia N, Dolk H (2018). Estimating Global Burden of Disease due to congenital anomaly: an analysis of European data. Arch Dis Child Fetal Neonatal Ed.

[18] Liu L, Johnson HL, Cousens S, Perin J, Scott S, Lawn JE, Rudan I, Campbell H, Cibulskis R, Li M, Mathers C, Black RE, Child Health Epidemiology Reference Group of WHO and UNICEF (2012). Global, regional, and national causes of child mortality: an updated systematic analysis for 2010 with time trends since 2000. Lancet.

[19] StataCorp (2019). Stata statistical software: release 16.

[20] Mazzucato M, Visonà Dalla Pozza L, Manea S, Minichiello C, Facchin P (2014). A population-based registry as a source of health indicators for rare diseases: the ten-year experience of the Veneto Region's rare diseases registry. Orphanet J Rare Dis.

[21] Longinetti E, Fang F (2019). Epidemiology of amyotrophic lateral sclerosis: an update of recent literature. Curr Opin Neurol.

[22] Kirkil G, Lower EE, Baughman RP (2018). Predictors of mortality in pulmonary sarcoidosis. Chest.

[23] Baican A, Chiorean R, Leucuta DC, Baican C, Danescu S, Ciuce D, Sitaru C (2015). Prediction of survival for patients with pemphigus vulgaris and pemphigus foliaceus: a retrospective cohort study. Orphanet J Rare Dis.

[24] Brick KE, Weaver CH, Lohse CM, Pittelkow MR, Lehman JS, Camilleri MJ, Al-Hashimi M, Wieland CN (2014). Incidence of bullous pemphigoid and mortality of patients with bullous pemphigoid in Olmsted County, Minnesota, 1960 through 2009. J Am Acad Dermatol.

[25] Kumar SK, Gertz MA, Lacy MQ, Dingli D, Hayman SR, Buadi FK, Short-Detweiler K, Zeldenrust SR, Leung N, Greipp PR, Lust JA, Russell SJ, Kyle RA, Rajkumar SV, Dispenzieri A (2011). Recent improvements in survival in primary systemic amyloidosis and the importance of an early mortality risk score. Mayo Clin Proc.

[26] Ciaramitaro P, Garbossa D, Peretta P, Piatelli G, Massimi L, Valentini L, Migliaretti G, Baldovino S, Roccatello D, Kodra Y, Taruscio D, Interregional Chiari and Syringomyelia Consortium; on behalf of the Interregional Chiari and Syringomyelia Consortium (2020). Syringomyelia and Chiari Syndrome Registry: advances in epidemiology, clinical phenotypes and natural history based on a North Western Italy cohort. Ann Ist Super Sanita.

[27] Gruber J, Saleh A, Bakhsh W, Rubery PT, Mesfin A (2018). The prevalence of Klippel–Feil syndrome: a computed tomography-based analysis of 2,917 patients. Spine Deform.

[28] Coi A, Barsotti S, Santoro M, Almerigogna F, Bargagli E, Caproni M, Emmi G, Frediani B, Guiducci S, Matucci Cerenic M, Mosca M, Parronchi P, Prediletto R, Selvi E, Simonini G, Tavoni AG, the Rare Diseases Working Group, Bianchi F, Pierini A (2021). Epidemiology of systemic sclerosis: a multi-database population-based study in Tuscany (Italy). Orphanet J Rare Dis.

[29] Ratanawatkul P, Solomon JJ, Kim D, George MP, Matarrese McGibbon LR, Demoruelle MK, Maleki-Fischbach M, Amigues I, Kastsianok L, Fernández Pérez ER (2020). Trends in systemic sclerosis and systemic sclerosis-related pulmonary arterial hypertension mortality in the USA. ERJ Open Res.

[30] Kodra Y, Posada de la Paz M, Coi A, Santoro M, Bianchi F, Ahmed F, Rubinstein YR, Weinbach J, Taruscio D (2017). Data quality in rare diseases registries. Adv Exp Med Biol.

