# Supplementary material for: Survival of patients with rare diseases: a population-based study in Tuscany (Italy)
Source: Orphanet J Rare Dis. 2021 Jun 14;16:275. doi: 10.1186/s13023-021-01907-0 (PMC8201697; doi:10.1186/s13023-021-01907-0)
Supplement: Supplementary file 1 — Additional file 1. Table S1. List of rare diseases endowed of an exemption code as defined by the Italian law and included in the study. For each nosological group, groups of diseases are reported in bold and, if available, specific diseases belonging to the groups are indicated. [file 13023_2021_1907_MOESM1_ESM.docx]

Table S1. List of rare diseases endowed of an exemption code as defined by the Italian law and included in the study.

| **INFECTIOUS AND PARASITIC DISEASES** | | | |
| --- | --- | --- | --- |
| *Disease and/or group* | *Specific diseases included in the group* | | |
| LEPROSY |  | | |
| LYME DISEASE |  | | |
| WHIPPLE DISEASE |  | | |
|  |  | | |
| **NEOPLASMS** | | | |
| *Disease and/or group* | *Specific diseases included in the group* | | |
| **CARNEY COMPLEX** |  | | |
| FAMILIAL ADENOMATOUS POLYPOSIS |  | | |
| GARDNER SYNDROME |  | | |
| GORLIN SYNDROME RETINOBLASTOMA |  | | |
| **HEREDITARY NONPOLYPOSIS COLON CANCER** | Lynch syndrome | | |
| LYMPHANGIOLEIOMYOMATOSIS |  | | |
| NEUROFIBROMATOSIS |  | | |
| NEPHROBLASTOMA |  | | |
| RETINOBLASTOMA |  | | |
|  |  | | |
| **ENDOCRINE DISEASES** | | | |
| *Disease and/or group* | *Specific diseases included in the group* | | |
| **ADRENOGENITAL SYNDROME** |  | | |
| **AUTOIMMUNE POLYENDOCRINOPATHY** | Schmidt's syndrome | | |
| CONGENITAL HYPOGONADOTROPIC HYPOGONADISM |  | | |
| CONGENITAL ISOLATED ACTH DEFICIENCY |  | | |
| FAMILIAL MEDULLARY THYROID CARCINOMA |  | | |
| IDIOPATHIC CENTRAL PRECOCIOUS PUBERTY |  | | |
| KALLMANN SYNDROME |  | | |
| LARON SYNDROME |  | | |
| LEPRECHAUNISM |  | | |
| MULTIPLE ENDOCRINE NEOPLASIA TYPE 1 |  | | |
| MULTIPLE ENDOCRINE NEOPLASIA TYPE 2A |  | | |
| NON-ACQUIRED ISOLATED GROWTH HORMONE DEFICIENCY |  | | |
| PENDRED SYNDROME |  | | |
| REFETOFF SYNDROME |  | | |
| **RARE PRIMARY HYPERALDOSTERONISM** | Primary hyperaldosteronism-seizures-neurological abnormalities syndrome  Primary unilateral adrenal hyperplasia | | |
|  |  | | |
| **METABOLIC DISEASES** | | | |
| *Disease and/or group* | *Specific diseases included in the group* | | |
| **DISORDERS OF MITOCHONDRIAL METABOLISM** | | | |
| **CREATINE DEFICIENCY SYNDROME** |  | | |
|  |  | | |
| KEARNS-SAYRE SYNDROME |  | | |
| **ISOLATED OXIDATIVE PHOSPHORYLATION COMPLEX DISORDER** |  | | |
| LEIGH SYNDROME |  | | |
| LEBER OPTIC ATROPHY |  | | |
| LONG CHAIN 3-HYDROXYACYL-COA DEHYDROGENASE DEFICIENCY |  | | |
| MEDIUM CHAIN ACYL-COA DEHYDROGENASE DEFICIENCY |  | | |
| MELAS |  | | |
| MERFF |  | | |
| **MITOCHONDRIAL OXIDATIVE PHOSPHORYLATION DISORDER DUE TO MITOCHONDRIAL DNA ANOMALIES** |  | | |
| **MITOCHONDRIAL OXIDATIVE PHOSPHORYLATION DISORDER DUE TO NUCLEAR DNA ANOMALIES** |  | | |
| **OTHER DISORDER OF MITOCHONDRIAL METABOLISM** | Epileptic encephalopathy with global cerebral demyelination | | |
| PYRUVATE DEHYDROGENASE PHOSPHATASE DEFICIENCY |  | | |
| SHORT CHAIN ACYL-COA DEHYDROGENASE DEFICIENCY |  | | |
| VERY LONG CHAIN ACYL-COA DEHYDROGENASE DEFICIENCY |  | | |
|  |  | | |
| **LYSOSOMAL STORAGE DISEASES** | | | |
| GALACTOSIALIDOSIS |  | | |
| **GANGLIOSIDOSIS** |  | | |
| KRABBE DISEASE |  | | |
| **LIPID STORAGE DISEASE** | Fabry disease  Gaucher disease  Niemann-Pick disease  Niemann-Pick disease type C | | |
| METACHROMATIC LEUKODYSTROPHY |  | | |
| MUCOLIPIDOSIS |  | | |
| **MUCOPOLYSACCHARIDOSIS** | Mucopolysaccharidosis type 4 | | |
| **NEURONAL CEROID LIPOFUSCINOSIS** |  | | |
| **OTHER LYSOSOMAL STORAGE DISEASES** | Glycogen storage disease due to LAMP-2 deficiency | | |
|  |  | | |
| **DISORDERS OF VITAMIN AND NON-PROTEIN COFACTOR ABSORPTION AND TRANSPORT** | | | |
| BIOTINIDASE DEFICIENCY |  | | |
| **DISORDER OF COBALAMIN AND FOLATE METABOLISM AND TRANSPORT** | Methylmalonic acidemia with homocystinuria, type cblC | | |
| **DISORDER OF OTHER VITAMINS AND COFACTORS METABOLISM AND TRANSPORT***  *excludes Ataxia with vitamin E deficiency |  | | |
| HYPOCALCEMIC VITAMIN D-DEPENDENT RICKETS |  | | |
| HYPOPHOSPHATEMIC RICKETS |  | | |
|  |  | | |
| **DISORDERS OF METAL METABOLISM AND TRANSPORT** | | | |
| ACERULOPLASMINEMIA |  | | |
| **DISORDER OF IRON METABOLISM AND TRANSPORT** | Rare hereditary hemochromatosis  Hereditary hyperferritinemia-cataract syndrome | | |
| MENKES DISEASE |  | | |
| WILSON DISEASE |  | | |
|  |  | | |
| **DISORDERS OF PROTEIN METABOLISM AND TRANSPORT** | | | |
| **CONGENITAL DISORDER OF GLYCOSYLATION** |  | | |
| CRIGLER-NAJJAR SYNDROME |  | | |
| **PRIMARY SYSTEMIC AMYLOIDOSIS** | Wild type ATTR amyloidosis | | |
|  |  | | |
| **OTHER METABOLIS DISEASES** | | | |
| ADIPOSIS DOLOROSA |  | | |
| ADRENOLEUKODYSTROPHY |  | | |
| CEREBROTENDINOUS XANTHOMATOSIS |  | | |
| **DISORDER OF AMINO ACID ABSORPTION AND TRANSPORT** | Albinism  Alkaptonuria  Cystinosis  Cystinuria  Glutaric aciduria  Glycine encephalopathy  Hyperprolinemia  Homocystinuria  Maple syrup urine disease  Methylmalonic acidemia  Phenylketonuria/ Hyperphenylalaninemia due to tetrahydrobiopterin deficiency  Pyruvate dehydrogenase E1-alpha deficiency  Tyrosinemia  Other acidemias or primitive organic acidurias from metabolism defects of branched chain amino acids | | |
| **DISORDER OF CARBOHYDRATE METABOLISM***  *excludes Diabetes mellitus | Fructose-1,6-bisphosphatase deficiency  Glucose transport disorder  Galactosemia  Glycogen storage disease  Glycogen storage disease due to acid maltase deficiency  Hereditary fructose intolerance | | |
| **DISORDER OF LIPID METABOLISM***  *excludes Combined familial hyperlipidemia, Dysbetalipoproteinemia, Heterozigous familial hypercholesterolemia type IIA and IIB, Polygenic hypercholesterolemia | Familial lipoprotein lipase deficiency  Homozygous familial hypercholesterolemia  Familial chylomicronemia syndrome Hypobetalipoproteinemia  Tangier disease | | |
| DISORDER OF NEUROTRANSMITTER AND PEPTIDE METABOLISM |  | | |
| **DISORDER OF PURINE OR PYRIMIDINE METABOLISM** | Lesch-Nyhan syndrome | | |
| **DISORDER OF UREA CYCLE METABOLISM AND AMMONIA DETOXIFICATION** | Citrullinemia  Ornithine transcarbamylase deficiency | | |
| FAMILIAL TUMORAL CALCINOSIS |  | | |
| **FAMILIAL HYPERINSULINISM** |  | | |
| GENERALIZED LIPODYSTROPHY |  | | |
| HYPOPHOSPHATASIA |  | | |
| **PEROXISOMAL DISEASE** |  | | |
| PORPHYRIA |  | | |
| REFSUM DISEASE |  | | |
| ZELLWEGER SYNDROME |  | | |
|  |  | | |
| **IMMUNE SYSTEM DISORDERS** | | | |
| *Disease and/or group* | *Specific diseases included in the group* | | |
| ACQUIRED ANGIOEDEMA WITH C1INH DEFICIENCY |  | | |
| ALPHA-1-ANTITRYPSIN DEFICIENCY |  | | |
| **AUTOINFLAMMATORY SYNDROME** | Periodic fever syndrome  Hyperimmunoglobulinemia D with periodic fever  Cryopyrin-associated periodic syndrome | | |
| **CHRONIC HISTIOCYTOSIS** | Erdheim-Chester disease  Langerhans cell histiocytosis  Histiocytosis X | | |
| FAMILIAL MEDITERRANEAN FEVER |  | | |
| HEREDITARY ANGIOEDEMA |  | | |
| NEONATAL ANTIPHOSPHOLIPID SYNDROME |  | | |
| **PRIMARY IMMUNODEFICIENCY** | Agammaglobulinemia  DiGeorge syndrome | | |
| SCHNITZLER SYNDROME |  | | |
| TUMOR NECROSIS FACTOR RECEPTOR 1 ASSOCIATED PERIODIC SYNDROME |  | | |
|  |  | | |
| **DISEASES OF THE BLOOD AND BLOOD-FORMING ORGANS** | | | |
| *Disease and/or group* | *Specific diseases included in the group* | | |
| CYCLIC NEUTROPENIA |  | | |
| CHRONIC GRANULOMATOUS DISEASE |  | | |
| **CHRONIC PRIMARY PLATELET DISORDER** | Immune thrombocytopenia | | |
| HEMOLYTIC UREMIC SYNDROME |  | | |
| **HEREDITARY ANEMIA***  *excludes Class I glucose-6-phosphate dehydrogenase deficiency | Blackfan-Diamond anemia  Fanconi anemia  Hereditary spherocytosis  Sickle cell anemia  Sideroblastic anemia  Thalassemia | | |
| **INHERITED COAGULATION DISORDER** | Hemophilia A  Hemophilia B  Rare hemorrhagic disorder due to a constitutional coagulation factors defect  Rare hereditary thrombophilia  Von Willebrand disease | | |
| **MYELODYSPLASTIC SYNDROME** | Refractory anemia | | |
| PAROXYSMAL NOCTURNAL HEMOGLOBINURIA |  | | |
| RARE ACQUIRED APLASTIC ANEMIA*  *excludes Transitory medullary aplasia |  | | |
| **RARE HEMORRHAGIC DISORDER DUE TO A CONSTITUTIONAL PLATELET ANOMALY** |  | | |
| **RARE HEMORRHAGIC DISORDER DUE TO A CONSTITUTIONAL THROMBOCYTOPENIA** |  | | |
| **SEVERE CONGENITAL NEUTROPENIA** | Adult idiopathic neutropenia | | |
| SHWACHMAN-DIAMOND SYNDROME |  | | |
| SYSTEMIC MASTOCYTOSIS |  | | |
|  |  | | |
| **PERIPHERAL AND CENTRAL NERVOUS SYSTEM DISORDERS** | | | |
| *Disease and/or group* | *Specific diseases included in the group* | | |
| AMYOTROPHIC LATERAL SCLEROSIS |  | | |
| **AUTOSOMAL DOMINANT CEREBELLAR ATAXIA** | Ataxia with vitamin E deficiency  Cerebellar ataxia-hypogonadism syndrome  Fragile X-associated tremor/ataxia syndrome  Friedreich ataxia  Hereditary episodic ataxia  Hereditary spastic paraplegia  Marie's cerebellar ataxia  Marinesco-Sjögren syndrome  Pantothenate kinase-associated neurodegeneration | | |
| BILATERAL STRIOPALLIDODENTATE CALCINOSIS |  | | |
| CEREBRAL AUTOSOMAL DOMINANT ARTERIOPATHY-SUBCORTICAL INFARCTS-LEUKOENCEPHALOPATHY |  | | |
| CHRONIC INFLAMMATORY DEMYELINATING POLYNEUROPATHY |  | | |
| **CONGENITAL MYASTHENIC SYNDROME** | Myasthenia gravis | | |
| **CONGENITAL MYOPATHY** | Central core disease  Centronuclear myopathy  Nemaline myopathy  Qualitative or quantitative defects of desmin | | |
| DENTATORUBRAL PALLIDOLUYSIAN ATROPHY |  | | |
| DRAVET SYNDROME |  | | |
| FAMILIAL OR SPORADIC HEMIPLEGIC MIGRAINE |  | | |
| **GENETIC PERIPHERAL NEUROPATHY** | Charcot-Marie-Tooth disease/Hereditary motor and sensory neuropathy  Dejerine-Sottas syndrome  Giant axonal neuropathy  Hereditary neuropathy with liability to pressure palsies  Hereditary sensory and autonomic neuropathy  Roussy-Lévy syndrome | | |
| GUILLAIN-BARRÉ SYNDROME |  | | |
| HUNTINGTON DISEASE |  | | |
| IDIOPATHIC TORSION DYSTHONIA |  | | |
| ISAAC SYNDROME |  | | |
| LAMBERT-EATON MYASTHENIC SYNDROME |  | | |
| LANDAU-KLEFFNER SYNDROME |  | | |
| LENNOX-GASTAUT SYNDROME |  | | |
| **LEUKODYSTROPHY** | Aicardi-Goutières syndrome  Alexander disease  Pelizaeus-Merzbacher disease | | |
| MELKERSSON-ROSENTHAL SYNDROME |  | | |
| MULTIFOCAL MOTOR NEUROPATHY |  | | |
| MULTIPLE SYSTEM ATROPHY |  | | |
| **MUSCULAR DYSTROPHY** | Becker muscular dystrophy  Calpain-3-related limb-girdle muscular dystrophy R1  Duchenne muscular dystrophy  Facioscapulohumeral dystrophy  Oculogastrointestinal muscular dystrophy | | |
| **MYOTONIC DYSTROPHY** | Paramyotonia congenita of Von Eulenburg  Steinert myotonic dystrophy  Thomsen and Becker disease | | |
| NARCOLEPSY |  | | |
| **NEURODEGENERATION WITH BRAIN IRON ACCUMULATION** | Infantile neuroaxonal dystrophy | | |
| NEURONAL INTRANUCLEAR INCLUSION DISEASE |  | | |
| **PERIODIC PARALYSIS** |  | | |
| POEMS SYNDROME |  | | |
| **PRIMARY DYSTONIA** |  | | |
| PRIMARY LATERAL SCLEROSIS |  | | |
| PROGRESSIVE MYOCLONIC EPILEPSY |  | | |
| PROGRESSIVE SUPRANUCLEAR PALSY |  | | |
| RETT SYNDROME |  | | |
| RIBOFLAVIN TRANSPORTER DEFICIENCY |  | | |
| **SPINAL MUSCULAR ATROPHY** | Kennedy disease  Proximal spinal muscular atrophy type 1  Proximal spinal muscular atrophy type 3 | | |
| STIFF PERSON SPECTRUM DISORDER |  | | |
| SYRINGOMYELIA |  | | |
| WEST SYNDROME |  | | |
|  |  | | |
| **DISORDERS OF THE EYE AND ADNEXA** | | | |
| *Disease and/or group* | *Specific diseases included in the group* | | |
| COGAN SYNDROME |  | | |
| CONGENITAL STATIONARY NIGHT BLINDNESS |  | | |
| **CORNEAL DYSTROPHY** | Epithelial basement membrane dystrophy  Fuchs endothelial corneal dystrophy  Granular corneal dystrophy type II  Macular corneal dystrophy  Meesmann corneal dystrophy  Posterior polymorphous corneal dystrophy  Reis-Bücklers corneal dystrophy | | |
| FAMILIAL EXUDATIVE VITREORETINOPATHY |  | | |
| **ISOLATED CHORIORETINAL DYSTROPHY** |  | | |
| **ISOLATED INHERITED RETINAL DISORDER** | Best vitelliform macular dystrophy  Leber congenital amaurosis  Progressive cone dystrophy  Retinitis pigmentosa  Retinitis punctata albescens  Stargardt disease  Vitreoretinal distrophy | | |
| OGUCHI DISEASE |  | | |
| POSTERIOR UVEITIS |  | | |
| SERPIGINOUS CHOROIDITIS |  | | |
| SYNDROMIC KERATOCONUS |  | | |
|  |  | | |
| **CIRCULATORY SYSTEM DISEASES** | | | |
| *Disease and/or group* | *Specific diseases included in the group* | | |
| ANTI-GLOMERULAR BASEMENT MEMBRANE DISEASE |  | | |
| BEHÇET DISEASE |  | | |
| BUDD-CHIARI SYNDROME |  | | |
| CRYOGLOBULINEMIC VASCULITIS |  | | |
| EOSINOPHILIC GRANULOMATOSIS WITH POLYANGIITIS |  | | |
| GIANT CELL ARTERITIS |  | | |
| GRANULOMATOSIS WITH POLYANGIITIS |  | | |
| HEREDITARY HEMORRHAGIC TELANGIECTASIA |  | | |
| IMMUNOGLOBULIN A VASCULITIS |  | | |
| MICROSCOPIC POLYANGIITIS |  | | |
| POLYARTERITIS NODOSA |  | | |
| **PRIMARY LYMPHEDEMA** | Milroy disease  Meige disease  Idiopathic primary lymphedema  Recessive primary congenital lymphedema | | |
| RHEUMATIC HEART DISEASE |  | | |
| TAKAYASU ARTERITIS |  | | |
| **THROMBOTIC MICROANGIOPATHY** | Hemolytic uremic syndrome  Thrombotic thrombocytopenic purpura | | |
|  |  | | |
| **RESPIRATORY DISEASES** | | | |
| *Disease and/or group* | *Specific diseases included in the group* | | |
| AUTOIMMUNE PULMONARY ALVEOLAR PROTEINOSIS |  | | |
| IDIOPATHIC PULMONARY ARTERIAL HYPERTENSION |  | | |
| IDIOPATHIC PULMONARY HEMOSIDEROSIS |  | | |
| ONDINE SYNDROME |  | | |
| PRIMARY CILIARY DYSKINESIA |  | | |
| PRIMARY CILIARY DYSKINESIA, KARTAGENER TYPE |  | | |
| **PRIMARY INTERSTITIAL LUNG DISEASE IN CHILDHOOD AND ADULTHOOD** | Acute interstitial pneumonia  Idiopathic pulmonary fibrosis | | |
| SARCOIDOSIS |  | | |
|  |  | | |
| **DIGESTIVE DISORDERS** | | | |
| *Disease and/or group* | *Specific diseases included in the group* | | |
| CHRONIC INTESTINAL PSEUDOOBSTRUCTION |  | | |
| CONGENITAL CHLORIDE DIARRHEA |  | | |
| EOSINOPHILIC GASTROENTERITIS |  | | |
| **ISOLATED ACHALASIA AND SYNDROMES-ASSOCIATED ACHALASIA** | Triple A syndrome | | |
| PRIMARY INTESTINAL LYMPHANGIECTASIA |  | | |
| PRIMARY SCLEROSING CHOLANGITIS |  | | |
| **PROGRESSIVE FAMILIAL INTRAHEPATIC CHOLESTASIS** | Progressive familial intrahepatic cholestasis type 3 | | |
|  |  | | |
|  |  | | |
| **DISEASES OF THE GENITOURINARY SYSTEM** | | | |
| *Disease and/or group* | *Specific diseases included in the group* | | |
| ALPORT SYNDROME |  | | |
| IGG4-RELATED RETROPERITONEAL FIBROSIS |  | | |
| INTERSTITIAL CYSTITIS |  | | |
| NEPHROGENIC DIABETES INSIPIDUS |  | | |
| **PRIMARY GLOMERULAR DISEASE** |  | | |
| **RARE RENAL TUBULAR DISEASE** | Bartter syndrome  Dent disease  Gitelman syndrome | | |
|  |  | | |
| **DISEASES OF THE SKIN AND SUBCUTANEOUS TISSUE** | | |  |
| *Disease and/or group* | *Specific diseases included in the group* | | |
| APLASIA CUTIS CONGENITA |  | | |
| BULLOUS PEMPHIGOID |  | | |
| CONGENITAL NON-BULLOUS ICHTHYOSIFORM ERYTHRODERMA |  | | |
| CUTIS LAXA |  | | |
| DARIER DISEASE |  | | |
| DIFFUSE CUTANEOUS SYSTEMIC SCLEROSIS |  | | |
| DYSKERATOSIS CONGENITA |  | | |
| EEC SYNDROME |  | | |
| **ECTODERMAL DYSPLASIA SYNDROME** |  | | |
| EPIDERMAL NEVUS SYNDROME |  | | |
| ERYTHROKERATODERMIA VARIABILIS |  | | |
| FAMILIAL ATYPICAL MULTIPLE MOLE MELANOMA SYNDROME |  | | |
| **HEREDITARY PALMOPLANTAR KERATODERMA** |  | | |
| HYPOMELANOSIS OF ITO |  | | |
| IBIDS SYNDROME |  | | |
| INCONTINENTIA PIGMENTI |  | | |
| INHERITED EPIDERMOLYSIS BULLOSA |  | | |
| **INHERITED ICHTHYOSIS** | Autosomal recessive congenital ichthyosis  Lamellar ichthyosis  Netherton syndrome  X-linked ichthyosis | | |
| KERATOSIS FOLLICULARIS SPINULOSA DECALVANS |  | | |
| LICHEN SCLEROSUS ET ATROPHICUS |  | | |
| MUCOUS MEMBRANE PEMPHIGOID |  | | |
| PEMPHIGUS |  | | |
| PSEUDOXANTHOMA ELASTICUM |  | | |
| PYODERMA GANGRENOSUM |  | | |
| SJÖGREN-LARSSON SYNDROME |  | | |
|  |  | | |
| **DISEASES OF THE MUSCULOSKELETAL SYSTEM AND CONNECTIVE TISSUE** | | |  |
| *Disease and/or group* | *Specific diseases included in the group* | | |
| ANTISYNTHETASE SYNDROME |  | | |
| DERMATOMYOSITIS |  | | |
| DIFFUSE FASCIITIS |  | | |
| EOSINOPHILIC FASCIITIS |  | | |
| FIBRODYSPLASIA OSSIFICANS PROGRESSIVA |  | | |
| GORHAM-STOUT DISEASE |  | | |
| INCLUSION BODY MYOSITIS |  | | |
| MIXED CONNECTIVE TISSUE DISEASE |  | | |
| POLYMYOSITIS |  | | |
| RELAPSING POLYCHONDRITIS |  | | |
| SAPHO SYNDROME |  | | |
| SYSTEMIC SCLEROSIS |  | | |
|  |  | | |
| **CONGENITAL ANOMALIES, CHROMOSOMAL ABERRATIONS AND GENETIC SYNDROMES** | | |  |
| *Disease and/or group* | *Specific diseases included in the group* | | |
| **CONGENITAL MALFORMATIONS OF THE NERVOUS SYSTEM** | | | |
| ARNOLD-CHIARI MALFORMATION |  | | |
| CEREBELLAR AGENESIS |  | | |
| GERSTMANN SYNDROME |  | | |
| ISOLATED OR SYNDROMIC HOLOPROSENCEPHALY |  | | |
| ISOLATED OR SYNDROMIC MICROCEPHALY |  | | |
| JOUBERT SYNDROME AND RELATED DISORDERS |  | | |
| LISSENCEPHALY |  | | |
| **SYNDROME WITH CORPUS CALLOSUM AGENESIS/DYSGENESIS AS A MAJOR FEATURE** | Dandy-Walker sydrome | | |
| WALKER-WARBURG SYNDROME |  | | |
| **OTHER SEVERE AND DISABLING SYNDROMES WITH A CENTRAL NERVOUS SYSTEM MALFORMATION AS A MAJOR FEATURE** |  | | |
|  |  | | |
| **CONGENITAL MALFORMATIONS OF THE EYE** | | |  |
| ANIRIDIA |  | | |
| AXENFELD-RIEGER SYNDROME |  | | |
| COLOBOMA OF THE OPTIC NERVE |  | | |
| **ISOLATED OR SYNDROMIC CONGENITAL OCULAR COLOBOMA** |  | | |
| **MICROPHTHALMIA-ANOPHTHALMIA-COLOBOMA** | Microphthalmia, Lenz type | | |
| PETERS ANOMALY |  | | |
| RIEGER ANOMALY |  | | |
| SEPTO-OPTIC DYSPLASIA SPECTRUM |  | | |
| VOGT-KOYANAGI-HARADA DISEASE |  | | |
|  |  | | |
| **ISOLATED OR SYNDROMIC CONGENITAL CRANIOFACIAL ANOMALIES** | |  |  |
| ACROCEPHALOSYNDACTYLY |  | | |
| ANTLEY-BIXLER SYNDROME |  | | |
| APERT SYNDROME |  | | |
| PFEIFFER SYNDROME |  | | |
| PIERRE ROBIN SYNDROME |  | | |
| **OTHER SEVERE AND DISABLING ANOMALIES OF SKULL AND FACE BONES, INTEGUMENTS AND MUCOSA** | Crouzon disease  Frontofacionasal dysplasia  Isolated craniosynostosis  Isolated or syndromic cleft palate | | |
|  |  | | |
| **CONGENITAL FACIAL ABNORMALITIES** | | |  |
| GOLDENHAR SYNDROME |  | | |
| MOEBIUS SYNDROME |  | | |
| SCHINZEL-GIEDION SYNDROME |  | | |
|  |  | | |
| **CONGENITAL LIMB MALFORMATIONS** | | |  |
| ADAMS-OLIVER SYNDROME |  | | |
| **ARTHROGRYPOSIS MULTIPLEX CONGENITA** |  | | |
| CONGENITAL ABSENCE OF UPPER ARM AND FOREARM WITH HAND PRESENT |  | | |
| FEMORAL-FACIAL SYNDROME |  | | |
| FREEMAN-SHELDON SYNDROME |  | | |
| POLAND SYNDROME |  | | |
|  |  | | |
| **CONGENITAL HEART DISEASE** | | |  |
| KLIPPEL-TRÉNAUNAY SYNDROME |  | | |
| **RARE CONGENITAL NON-SYNDROMIC HEART MALFORMATION***  *excludes Isolated atrial septal defect, Isolated pulmonary valve stenosis,  Isolated ventricular septal defect, Patent ductus arteriosus | Ebstein malformation of the tricuspid valve  Hypoplastic left heart syndrome | | |
| **OTHER SEVERE AND DISABLING CONGENITAL SYNDROMIC VASCULAR DISEASE** | CLOVES syndrome  Capillary malformation-arteriovenous malformation | | |
|  |  | | |
| **ABDOMINAL WALL DEFECTS** | | |  |
| ISOLATED KLIPPEL-FEIL SYNDROME |  | | |
|  |  | | |
| **CONGENITAL ANOMALIES OF THE GASTROINTESTINAL TRACT** | | |  |
| ANORECTAL MALFORMATION |  | | |
| CAROLI DISEASE |  | | |
| DUPLICATION OF THE GASTROINTESTINAL TRACT |  | | |
| ESOPHAGEAL ATRESIA AND/OR ISOLATED TRACHEOESOPHAGEAL FISTULA |  | | |
| ISOLATED BILIARY ATRESIA |  | | |
| ISOLATED POLYCYSTIC LIVER DISEASE |  | | |
| HIRSCHSPRUNG DISEASE |  | | |
| PERSISTENT CLOACA |  | | |
|  |  | | |
| **CONGENITAL UROGENITAL ANOMALIES** | | |  |
| AUTOSOMAL RECESSIVE POLYCYSTIC KIDNEY DISEASE |  | | |
| BLADDER EXSTROPHY |  | | |
| **DISORDER OF SEXUAL DEVELOPMENT AND/OR AMBIGUOUS GENITALIA AND/OR KARIOTYPE DISCORDANCE/GONADAL DEVELOPMENT AND/OR PHENOTYPE** | Gonadal dysgenesis  Complete androgen insensitivity syndrome  Partial androgen insensitivity syndrome | | |
| MAYER-ROKITANSKY-KÜSTER-HAUSER SYNDROME |  | | |
| **FAMILIAL CYSTIC RENAL DISEASE** |  | | |
| MEDULLARY SPONGE KIDNEY |  | | |
| **PSEUDOHERMAPHRODITISM** |  | | |
| **OTHER SEVERE AND DISABLING DEFECTS OF SEXUAL DEVELOPMENT AND/OR AMBIGUOUS GENITALIA AND/OR KARIOTYPE DISCORDANCE/GONADAL DEVELOPMENT AND/OR PHENOTYPE** |  | | |
|  | | |  |
| **GENETIC SKELETAL DISORDERS** | | |  |
| ACRODYSOSTOSIS |  | | |
| **CONGENITAL CHONDRODISPLASIAS** | Achondroplasia  Jeune syndrome  Multiple osteochondromas | | |
| **CONGENITAL ISOLATED OR SYNDROMIC CHONDRODISPLASIAS** | Camurati-Engelmann disease  Craniometaphyseal dysplasia  Fibrous dysplasia  Léri-Weill dyschondrosteosis  Multiple epiphyseal dysplasia  McCune-Albright syndrome  Osteogenesis imperfecta  Osteopetrosis and related disorders  Spondyloepiphyseal dysplasia tarda | | |
| MAFFUCCI SYNDROME |  | | |
| SPONDYLOEPIPHYSEAL DYSPLASIA CONGENITA |  | | |
|  | | |  |
| **OTHER SYNDROMES AND COMPLEX CONGENITAL MALFORMATIONS** | | |  |
| 22q11.2 DELETION SYNDROME |  | | |
| AARSKOG-SCOTT SYNDROME |  | | |
| ALAGILLE SYNDROME |  | | |
| ALSTRÖM SYNDROME |  | | |
| ANGELMAN SYNDROME |  | | |
| BARDET-BIEDL SYNDROME |  | | |
| BECKWITH-WIEDEMANN SYNDROME |  | | |
| BOR SYNDROME |  | | |
| BORJESON-FORSSMAN-LEHMANN SYNDROME |  | | |
| BRANCHIO-OCULO-FACIAL SYNDROME |  | | |
| CARDIOFACIOCUTANEOUS SYNDROME |  | | |
| CHAR SYNDROME |  | | |
| CHARGE SYNDROME |  | | |
| COCKAYNE SYNDROME |  | | |
| COFFIN-SIRIS SYNDROME |  | | |
| COHEN SYNDROME |  | | |
| CORNELIA DE LANGE SYNDROME |  | | |
| DUBOWITZ SYNDROME |  | | |
| EHLERS-DANLOS SYNDROME |  | | |
| FRAGILE X SYNDROME |  | | |
| HOLT-ORAM SYNDROME |  | | |
| ISOLATED HEMIHYPERPLASIA |  | | |
| KABUKI SYNDROME |  | | |
| LOEYS-DIETZ SYNDROME |  | | |
| MARFAN SYNDROME |  | | |
| MARSHALL SYNDROME |  | | |
| MARSHALL-SMITH SYNDROME |  | | |
| MONOSOMY 5P |  | | |
| **MOSAIC VARIEGATED ANEUPLOIDY SYNDROME** |  | | |
| **MULTIPLE CONGENITAL ANOMALIES/DYSMORPHIC SYNDROME-INTELLECTUAL DISABILITY** | KBG syndrome | | |
| **MULTIPLE HAMARTOMAS** | Cowden syndrome  Hepatic cystic hamartoma | | |
| NAIL-PATELLA SYNDROME |  | | |
| NOONAN SYNDROME AND NOONAN-RELATED SYNDROME |  | | |
| NOONAN SYNDROME WITH MULTIPLE LENTIGINES |  | | |
| OCULODENTODIGITAL DYSPLASIA |  | | |
| OPITZ GBBB SYNDROME |  | | |
| PEUTZ-JEGHERS SYNDROME |  | | |
| PRADER-WILLI SYNDROME |  | | |
| PROGRESSIVE HEMIFACIAL ATROPHY |  | | |
| RUBINSTEIN-TAYBI SYNDROME |  | | |
| SALDINO-MAINZER SYNDROME |  | | |
| SECKEL SYNDROME |  | | |
| SILVER-RUSSELL SYNDROME |  | | |
| SMITH-MAGENIS SYNDROME |  | | |
| SOTOS SYNDROME |  | | |
| STICKLER SYNDROME |  | | |
| STURGE-WEBER SYNDROME |  | | |
| **SYNDROMIC CHROMOSOMAL AND GENOMIC UNBALANCED REARRANGEMENTS** | Distal 22q11.2 microdeletion syndrome | | |
| TETRASOMY 12P |  | | |
| TOWNES-BROCKS SYNDROME |  | | |
| TUBEROUS SCLEROSIS COMPLEX |  | | |
| TURNER SYNDROME |  | | |
| VACTERL/VATER ASSOCIATION |  | | |
| VON HIPPEL-LINDAU DISEASE |  | | |
| WAGR SYNDROME |  | | |
| **WAARDENBURG SYNDROME** |  | | |
| WILLIAMS SYNDROME |  | | |
| WOLF-HIRSCHHORN SYNDROME |  | | |
| WOLFRAM SYNDROME |  | | |
|  |  | | |
| **NEONATAL MORBITIES OF PERINATAL ORIGIN** | | |  |
| *Disease and/or group* | *Specific diseases included in the group* | | |
| BILIRUBIN ENCEPHALOPATHY |  | | |
| FETAL ALCOHOL SYNDROME |  | | |
| HEPATIC FIBROSIS-RENAL CYSTS-INTELLECTUAL DISABILITY SYNDROME |  | | |

For each nosological group, groups of diseases are reported in bold and, if available, specific diseases belonging to the groups are indicated.
